# Pannexin1 channels in the liver: an open enemy

**DOI:** 10.3389/fcell.2023.1220405

**Published:** 2023-07-10

**Authors:** Raf Van Campenhout, Anne Caufriez, Andrés Tabernilla, Amy Maerten, Sybren De Boever, Julen Sanz-Serrano, Prashant Kadam, Mathieu Vinken

**Affiliations:** Entity of In Vitro Toxicology and Dermato-Cosmetology, Department of Pharmaceutical and Pharmacological Sciences, Vrije Universiteit Brussel, Brussels, Belgium

**Keywords:** pannexin1, physiology, pathology, inflammation, cell death, liver disease

## Abstract

Pannexin1 proteins form communication channels at the cell plasma membrane surface, which allow the transfer of small molecules and ions between the intracellular compartment and extracellular environment. In this way, pannexin1 channels play an important role in various cellular processes and diseases. Indeed, a plethora of human pathologies is associated with the activation of pannexin1 channels. The present paper reviews and summarizes the structure, life cycle, regulation and (patho)physiological roles of pannexin1 channels, with a particular focus on the relevance of pannexin1 channels in liver diseases.

## 1 Introduction

Pannexin (Panx) proteins were first described in 2000. In the human body, 3 different types of Panx proteins are expressed, namely, Panx1, Panx2 and Panx3 (Vinken, 2022). Panx1 is undoubtedly the most abundantly studied Panx family member. Human Panx1 proteins are widely expressed and can be found in the brain, skin, lung, eye, breast, gastrointestinal tract, urinary system, reproductive system and blood cells (Table 1). These Panx1 proteins can assemble into oligomers to form communication channels (Deng et al., 2020; Qu et al., 2020; Zhang S. et al., 2021; Vinken, 2022). Over the past years, a large body of evidence has been generated showing that Panx1 channels are involved in physiological and pathological processes (Penuela et al., 2013; Vinken, 2022). Consequently, Panx1 channels are regarded as key determinants in the induction and propagation of inflammation and cell death processes, being hallmarks of a plethora of liver diseases (Cooreman et al., 2019; Vinken, 2022). Because of its unique anatomic location and multidimensional functions, the liver is a frequent target for disease (Vinken, 2020). Liver disease is one of the top 10 most common causes of death worldwide (Blachier et al., 2013; Ritchie et al., 2018). Liver disease is considered as a major public health concern affecting billions of people (Cheemerla and Balakrishnan, 2021; Riazi et al., 2022) and imposing a significant economic burden (Younossi et al., 2016). Moreover, it has become clear that the acting of Panx1 channels as so-called pathological pores equally holds true for hepatic Panx1 channels. In this review paper, we describe the structure, life cycle, regulation and (patho)physiological role of Panx1 channels. Critical attention is paid to the relevance of Panx1 channels in pathophysiology, in the specific context of acute and chronic liver disease.

**TABLE 1:** Human pannexin1 (Panx1) proteins are widely expressed. Panx1 proteins can be found in the brain, skin, lung, eye, breast, gastrointestinal tract, urinary system, reproductive system and blood cells.

Organ system	Tissue or cell type	Reference(s)
Nervous system	Cortical tissue	Dossi et al. (2018)
	Glioblastoma cells	Wei et al. (2015)
	Eye	Žužul et al. (2022)
	Corneal tissue	Cui et al. (2016)
Integumentary system	Skin and adnexal structures	Cowan et al. (2012)
	Epidermal sensory cells	Cárcaba et al. (2022)
	Breast epithelial cells	Jalaleddine et al. (2019)
Digestive system	Liver tissue	Leroy et al. (2022), Wang et al. (2022)
	Hepatocytes	Wang et al. (2022)
	Colon segments	Diezmos et al. (2013)
	Pancreatic islets	Bartley et al. (2019)
	Colonic carcinoma cells	Alhouayek et al. (2019)
	Gastric carcinoma cells	Ying et al. (2021)
Respiratory system	Lung tissue	Luu et al. (2021)
	Airway epithelial cells	Ransford et al. (2009)
	Tracheal epithelial cells	Ransford et al. (2009)
Urinary system	Kidney tissue	Jeličić et al. (2022)
	Bladder urethral cells	Negoro et al. (2013)
	Proximal tubular cells	Su et al. (2019)
Reproductive system	Uterus epithelial cells	Imamura et al. (2020)
	Umbilical vein endothelial cells	Lohman et al. (2015)
Circulatory system	Red blood cells	Rougé et al. (2022)
	Macrophages	Chen et al. (2019)
	Monocytes	Parzych et al. (2017), Chen et al. (2019)
	Thrombocytes	Li et al. (2023), Metz et al. (2023)
	T-lymphocytes	Boyd-Tressler et al. (2014)

## 2 Structure, life cycle, regulation and (patho)physiological roles of pannexin1 channels

### 2.1 Structure

**FIGURE 1:**
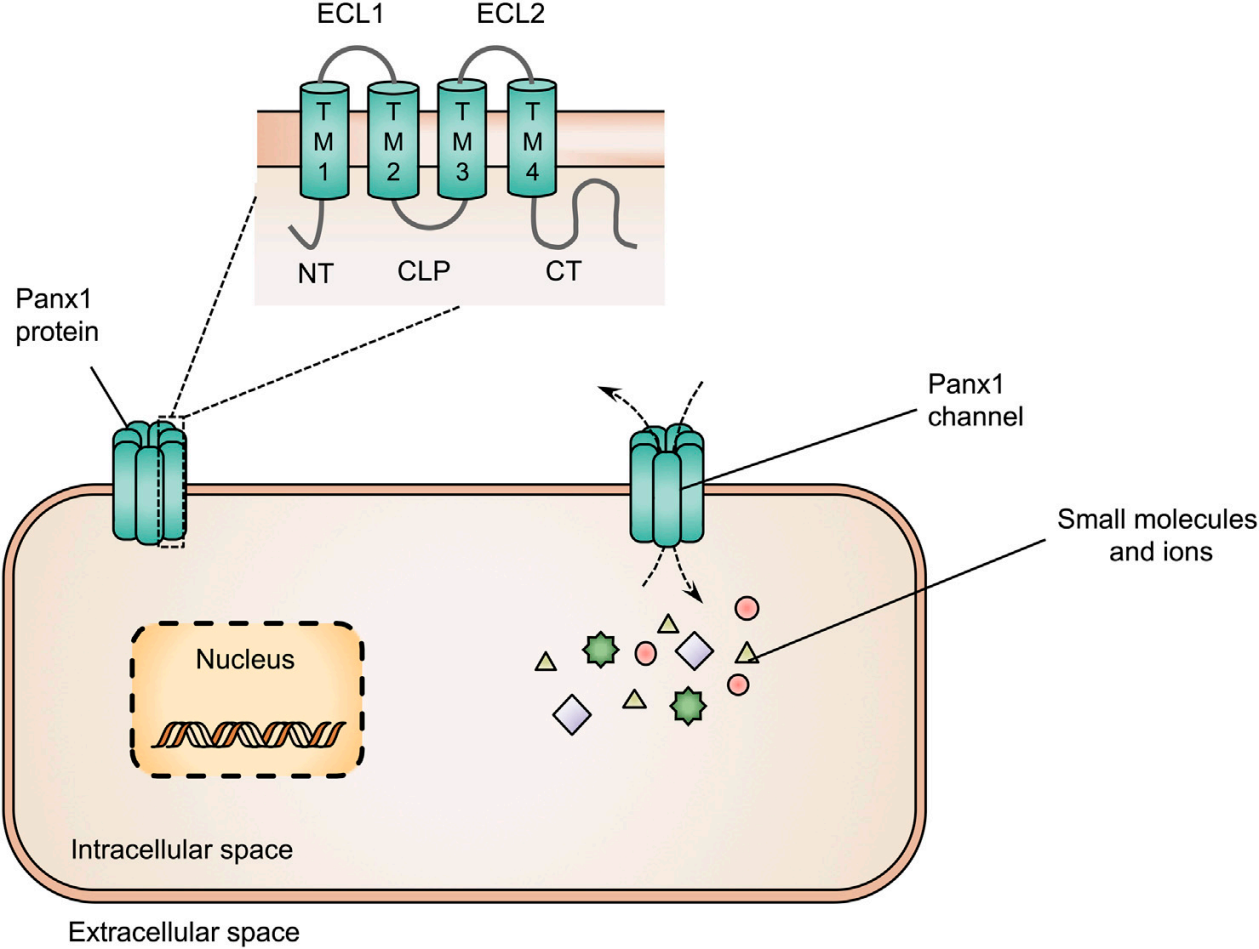
Structure of pannexin1 (Panx1) proteins and Panx1 channels. Panx1 proteins consist of 4 transmembrane domains (TM1-4), 2 extracellular loops (ECL1-2), 1 cytoplasmic loop (CLP), 1 cytoplasmic *N*-terminal (NT) and 1 cytoplasmic *C*-terminal tail (CT). Panx1 proteins form heptameric channels at the cell plasma membrane surface to mediate the transfer of small molecules and ions between the intracellular and extracellular space.

Panx1 proteins are multi-pass transmembrane proteins consisting of 4 transmembrane domains, 2 extracellular loops, 1 cytoplasmic loop, 1 cytoplasmic *N*-terminal and 1 cytoplasmic *C*-terminal tail (Laird and Penuela, 2021; Vinken, 2022) (Figure 1). These Panx1 proteins can build-up heptameric channels, which facilitate paracrine communication by mediating the transfer of small molecules, including adenosine triphosphate (ATP), cyclic adenosine monophosphate and inositol triphosphate, and ions, such as calcium, chloride and potassium. By doing so, Panx1 channels control cellular communication between the cytosol and extracellular environment (Figure 1) (Deng et al., 2020; Qu et al., 2020; Zhang S. et al., 2021; Vinken, 2022). Concerning the liver, the presence of Panx1 protein has been demonstrated in both human and mouse liver tissue (Penuela et al., 2007; Leroy et al., 2022). Analysis of hepatic Panx1 mRNA levels in mice revealed that Panx1 is expressed by both parenchymal and non-parenchymal cells. In particular, Panx1 RNA was found in hepatic stellate cells, hepatocytes and Kupffer cells but not in liver sinusoidal endothelial cells (Willebrords et al., 2018).

### 2.2 Life cycle

The *PANX1* gene is located on human chromosome 11q14.3, consisting of 5 exons and 4 introns (Baranova et al., 2004). As exon 5 is present in 2 variants, the human *PANX1* gene drives the generation of 2 isoforms, *PANX1a* and *PANX1b*. *PANX1a* mRNA corresponds to a 426 amino acid protein with a molecular weight of 47.6 kDa, whereas the *Panx1b* mRNA encodes for a variant with an insertion of a 4 amino acid sequence 22 amino acids upstream of the *C*-terminal tail (Baranova et al., 2004). Furthermore, 2 shorter *Panx1* isoforms, *Panx1c* and *Panx1d*, have been characterized in rat pituitary cells. Both are generated from alternative splicing at exons 2 and 4, respectively. *Panx1c* and *Panx1d* show a different cellular distribution pattern. Both splice variants are predominantly intracellular localized and they negatively modulate the functions of Panx1a through formation of heteromeric channels (Li et al., 2011). The presence of *Panx1* splice variants might be explained by the notion that the *Panx1* promotor is regulated via different mechanisms. The *Panx1* promotor, which is highly homologous between rat, mouse and human sequences, possesses multiple transcriptional starting sites. Transcription factors E26 transformation-specific translocation variant 4 (ETV4) and 3′,5′-cyclic adenosine monophosphate response element-binding protein (CREB) bind to the rat *Panx1* gene promoter to start transcription (Dufresne and Cyr, 2014). Next to cis/trans regulation, *Panx1* gene expression is controlled by epigenetic mechanisms. In particular, methylation of DNA (Dufresne and Cyr, 2014) and changes in histone modifications (Zhang et al., 2015) contribute to increased Panx1 expression levels.

### 2.3 Regulation

**FIGURE 2:**
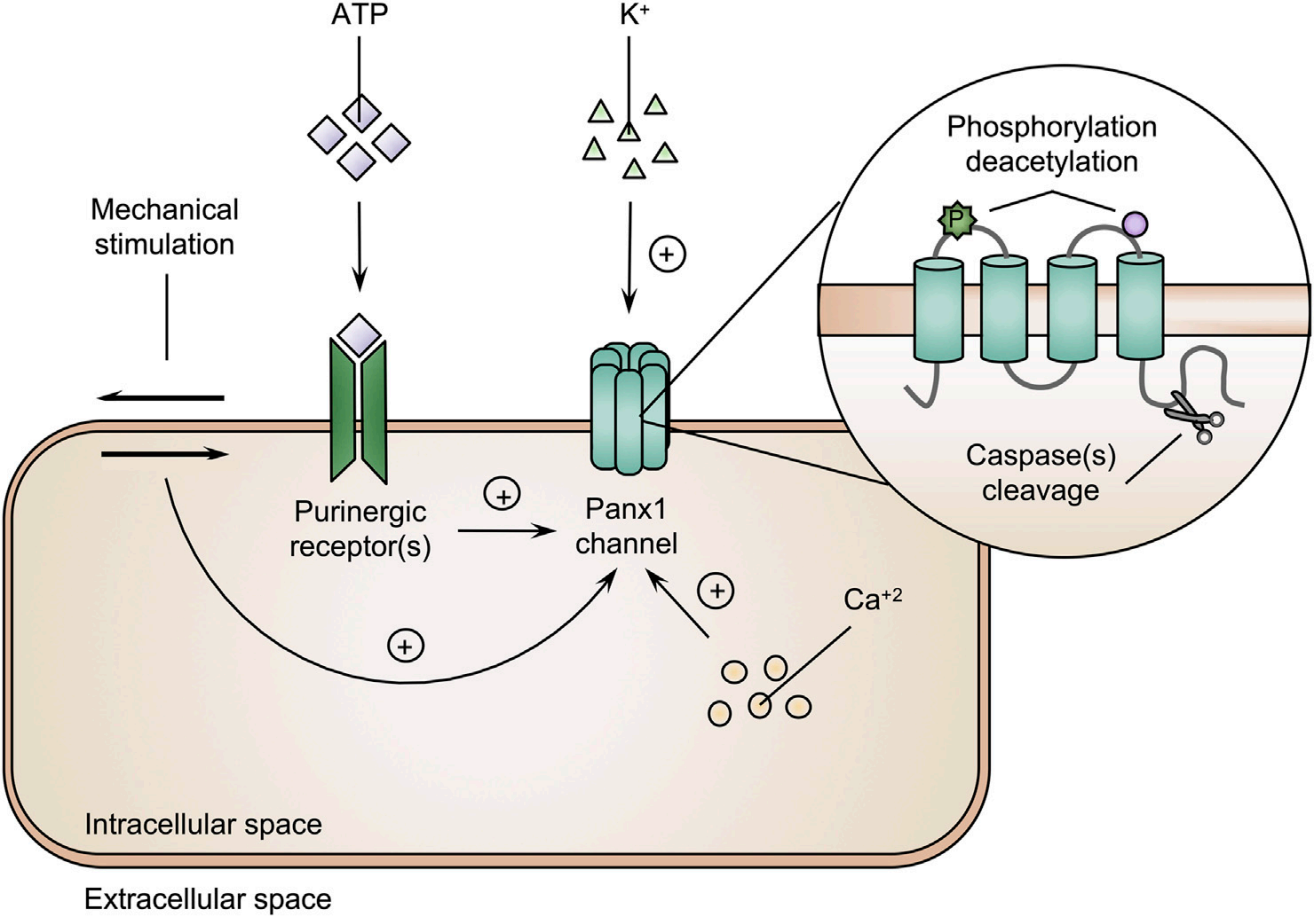
Pannexin1 (Panx1) channels are activated by various triggers. The amount of intracellular calcium ions (Ca^2+^), extracellular adenosine triphosphate (ATP) molecules and potassium (K^+^) ions, phosphorylation (P), deacetylation and cleavage of Panx1 proteins and mechanical stimulation are underlying mechanisms triggering Panx1 channel activity (+ = induction).

Panx1 proteins follow the classical endoplasmic reticulum-Golgi secretory pathway to form Panx1 channels at the cell plasma membrane surface (Bhalla-Gehi et al., 2010). While possibly serving as a reservoir of precursor Panx1 proteins, endoplasmic reticulum-bound Panx1 could equally reflect a role as a calcium channel (Vanden Abeele et al., 2006). Differences in subcellular localization are due to post-translational modifications. Unglycosylated Panx1 (Gly0) becomes glycosylated in the endoplasmic reticulum resulting in the high-mannose status (Gly1) (Penuela et al., 2007). Subsequently, Panx1 moves to the Golgi apparatus, where it is subjected to another round of glycosylation, yielding the Gly2 variant. This glycosylation event is involved in the trafficking to the cell plasma membrane surface (Penuela et al., 2007; Bhalla-Gehi et al., 2010). Accordingly, the Gly2 form is more abundant at the cell plasma membrane, while the Gly0 and Gly1 variants are preferentially found in the endoplasmic reticulum (Penuela et al., 2007). Cell plasma membrane-associated Panx1 proteins exhibit a fixed expression profile, *i.e.*, their cell surface localization is steady for at least 8 h, after which they are internalized and subjected to lysosomal degradation (Penuela et al., 2007; Boassa et al., 2008; Bhalla-Gehi et al., 2010; Gehi et al., 2011). At the cell plasma membrane surface, Panx1 channels are activated by various triggers, including mechanical stress, intracellular calcium ions, extracellular ATP molecules and potassium ions (Qiu and Dahl, 2009; Suadicani et al., 2012; Diem et al., 2020; Lee et al., 2022). Furthermore, Panx1 channels are modulated by post-translational modifications of Panx1 proteins, including phosphorylation, deacetylation and proteolysis (Figure 2). First, the phosphorylation-induced channel activity is evidenced by conformational changes in the Panx1 channel structure, which increasing channel permeability *in vitro* (López et al., 2020). Moreover, kinase inhibitors are able to block Panx1 channel-mediated ATP release, indicating the involvement of tyrosine kinases in channel activity (Weilinger et al., 2012; Weilinger et al., 2016; DeLalio et al., 2019; Lohman et al., 2019). Second, acetylation/deacetylation reactions regulate Panx1 channel activity. Thus, histone deacetylase 6 evokes the opening of Panx1 channels *in vitro* (Chiu et al., 2021). Third, Panx1 channels are persistently activated by caspase-mediated cleavage of the *C*-terminal region of Panx1 proteins (Sandilos et al., 2012). Such caspase-driven channel activation can be mediated by both caspase 3 and caspase 11 (Yang et al., 2015; Narahari et al., 2021).

### 2.4 Physiological functions

Panx1 channels support the transfer of biomolecules and ions that play essential roles in many physiological processes (Table 2). Therefore, the ability of Panx1 channels to support the export of ATP molecules is critical to control hemodynamic responses. Panx1 channels modulate the vascular tone as evidenced by impairment of vasodilatory responses to hypoxia for *Panx1* knock-out mice. Panx1 channel-regulated export of ATP from red blood cells to plasma is essential to support changes in mean arterial pressure and hindlimb blood flow during hypoxia (Kirby et al., 2021). Panx1 channel-mediated ATP release also contributes to metabolic processes. Adipocytes indeed express functional Panx1 channels that regulate insulin-stimulated glucose uptake through the liberation of ATP (Adamson et al., 2015). In the central nervous system, ATP release through Panx1 channels is critical for hippocampus-dependent memory. Adult mice bearing loss of Panx1 channel activity indeed have increased postsynaptic excitability in the hippocampal region (Prochnow et al., 2012; Ardiles et al., 2014; Südkamp et al., 2021). Ablation of Panx1 proteins gives rise to comprised synaptic plasticity, substantiated by impairment of spatial and object recognition memory in *Panx1* knock-out mice (Prochnow et al., 2012). Panx1 channel-mediated ATP transport is also required for hearing (Chen et al., 2015). Thus, Panx1 channels located in the cochlear wall of mice release ATP to ensure endocochlear and auditory receptor potential generation (Chen et al., 2015). Furthermore, physiological functions for Panx1 proteins have been proposed in the eye. Panx1 channel activity influences retinal signal transmission mechanisms, regulates microglial morphology and controls dynamic behavior (Fontainhas et al., 2011; Kranz et al., 2013; Kurtenbach et al., 2014). Genetic ablation of *Panx1* in mice revealed a prominent function for Panx1 channels in skeletal muscle regeneration. Myoblasts derived from *Panx1* knock-out animals display low migratory characteristics, morphological changes and more prominent surface blebs compared to wild-type counterparts. This deletion of *Panx1* goes hand in hand with disturbed myoblast functioning, which is reflected by deficits in muscle regeneration (Suarez-Berumen et al., 2021). Moreover, Panx1 channel activity promotes mucociliary lung clearance (Seminario-Vidal et al., 2011; van Heusden et al., 2021). Panx1 channel-mediated ATP release contributes to normal lung physiology, since ciliary beat frequencies and mucociliary clearance activities are lower upon deletion of *Panx1* in mice (van Heusden et al., 2021). Collectively, these data show that Panx1 channel activity underlies various physiological functions. The clinical relevance of these findings is demonstrated by a case of a 17-year-old female bearing a homozygous germline variant in the *PANX1* gene. Expression of a R217H Panx1 variant is associated with loss of channel functionality, and provokes dysfunctionalities in multiple organs, including primary ovarian failure, intellectual disability, sensorineural hearing loss and skeletal defects (Shao et al., 2016).

**TABLE 2:** Role of pannexin1 (Panx1) channels in physiological processes.

Physiological process	Tissue or cell type	Species	Panx1 channel activity	Effect	Reference(s)
Hemodynamic responses	Red blood cells	Mouse	Extracellular ATP release	Supports changes in mean arterial pressure and hindlimb blood flow during hypoxia	Kirby et al. (2021)
Metabolic processes	Adipocytes	Mouse	Extracellular ATP release	Regulates insulin-stimulated glucose uptake	Adamson et al. (2015)
Hippocampus-dependent memory	CA1 pyramidal neurons	Mouse	Extracellular ATP release	Controls postsynaptic excitability	Prochnow et al. (2012), Ardiles et al. (2014), Südkamp et al. (2021)
Hearing	Cochlea	Mouse	Extracellular ATP release	Ensures endocochlear and auditory receptor potential generation	Chen et al. (2015)
Neurotransmission signaling	Retinal microglia cells	Mouse	Extracellular ATP release	Influences retinal signal transmission mechanisms, regulates microglial morphology and controls dynamic behavior	Fontainhas et al. (2011)
Skeletal muscle regeneration	Myoblasts	Mouse	Extracellular ATP release	Supports myoblast functioning	Suarez-Berumen et al. (2021)
Skeletal muscle contraction	Muscle fibers	Rat	Extracellular ATP release	Potentiation of muscle contraction	Riquelme et al. (2013)
Normal lung physiology	Tracheal epithelial cells	Mouse	Extracellular ATP release	Mediate ciliary beat frequencies and mucociliary clearance activities	van Heusden et al. (2021)

### 2.5 Pathological functions

#### 2.5.1 Inflammation

##### 2.5.1.1 Inflammasome signaling

**FIGURE 3:**
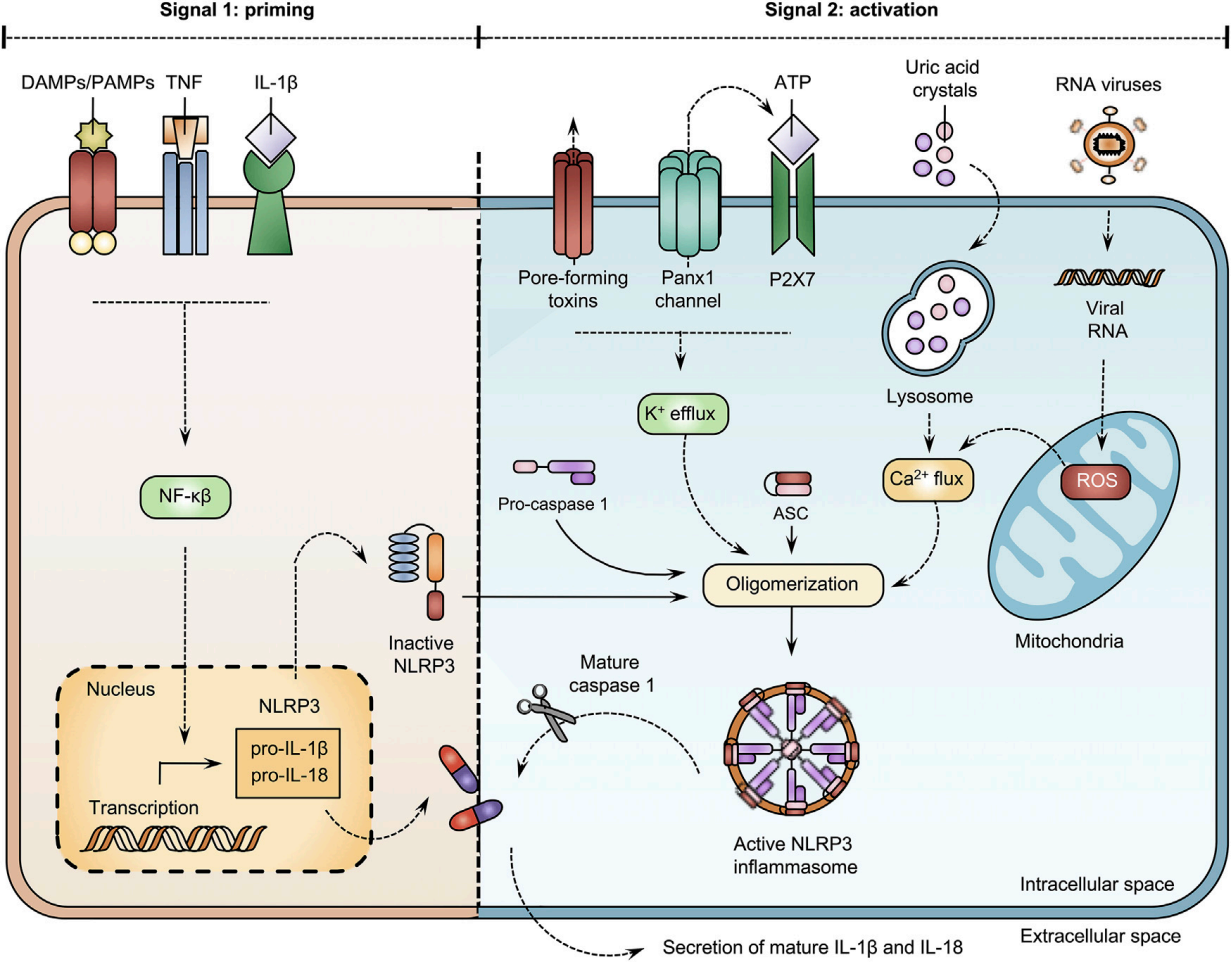
Open pannexin1 (Panx1) channels stimulate NOD-like receptor family pyrin domain containing 3 (NLRP3) inflammasome activation. Canonical activation of the NLRP3 inflammasome generally requires 2 signals. During the priming step, damage-associated molecular patterns (DAMPs), pathogen-associated molecular patterns (PAMPs) and cytokines, including, tumor necrosis factor (TNF) and interleukin (IL)-1β activate the nuclear factor (NF)-κβ transcription factor. Consequently, NF-κβ-dependent gene transcription induces the expression of NLRP3, pro-IL-1β and pro-IL-18. The second signal requires the presence of NLRP3 agonists. Extracellular adenosine triphosphate (ATP) molecules, uric acid crystals and viral RNAs drive changes in the intracellular environment, including efflux of potassium, reactive oxygen species (ROS) formation and mobilization of calcium ions to stimulate oligomerization of NLRP3 with an apoptosis-associated speck-like protein containing caspase (ASC) and pro-caspase 1. Following assemblage, the NLRP3 inflammasome becomes functional and activates caspase 1, which drives the secretion of IL-1β and IL-18.

Inflammation is a complex, yet well-organized body response that can be mediated by inflammasomes, which are multiprotein complexes (Guo et al., 2015; Wang and Hauenstein, 2020). To activate the innate immune system, pathogen-associated molecular patterns (PAMPs) and damage-associated molecular patterns (DAMPs) bind to Toll-like receptors (TLRs) (Guo et al., 2015; Shao et al., 2015; Wang and Hauenstein, 2020; Unterberger et al., 2021). Upon TLR activation, inflammasomes regulate the maturation and production of interleukins (ILs). The NOD-like receptor family pyrin domain containing 3 (NLRP3) inflammasome, the most intensively studied inflammasome, is involved in many events related to activation and regulation of inflammation (Kelley et al., 2019; Zheng et al., 2020). Canonical activation of NLRP3 activity evokes the release of IL-1β and IL-18 and generally requires 2 signals (Figure 3). The first signal is denoted the priming step. During this step, DAMPs, PAMPs and cytokines, including tumor necrosis factor (TNF) and IL-1β, activate the nuclear factor (NF)-κβ transcription factor. Consequently, NF-κβ-dependent gene transcription induces the expression of NLRP3, pro-IL-1β and pro-IL-18 (Guo et al., 2015; Shao et al., 2015; Gritsenko et al., 2020; Wang and Hauenstein, 2020). The second signal requires the presence of NLRP3 agonists. Extracellular ATP molecules, uric acid crystals and viral RNAs drive changes in the intracellular environment, including efflux of potassium ions, reactive oxygen species (ROS) formation and mobilization of calcium ions to stimulate the oligomerization of NLRP3 with an apoptosis-associated speck-like protein containing caspase (ASC)-like protein and pro-caspase 1. Following assemblage, the NLRP3 inflammasome becomes functional and activates caspase 1, which drives the secretion of IL-1β and IL-18 (Guo et al., 2015; Shao et al., 2015; Kelley et al., 2019; Wang and Hauenstein, 2020). Panx1 channels act as key players in this NLRP3 inflammasome cascade. In particular, Panx1 channel-mediated ATP release triggers P2X7 receptors (Pelegrin and Surprenant, 2006; Kanneganti et al., 2007; Albalawi et al., 2017; Parzych et al., 2017). This exacerbates the efflux of potassium ions and perpetuates the opening of Panx1 channels (Parzych et al., 2017). Interplay between Panx1 channel and P2X7 receptor activity drives NLRP3 oligomerization, caspase 1 activation and secretion of ILs. This interaction is elicited by TLR stimulation (Parzych et al., 2017). Experiments with human acute monocytic leukemia THP-1 cells show that both TLR2 and TLR4 signaling induce activation of the NLRP3 inflammasome and subsequent IL-1β release. However, a differential role for Panx1 proteins is observed. While TLR2-mediated IL-1β release is induced through Panx1 channel and P2X7 receptor activation, TLR4 signaling is independent of Panx1 and P2X7 proteins (Parzych et al., 2017). Furthermore, a mechanism for cleavage-based activation of Panx1 channels underlying NLRP3 signaling has been identified in rat renal tubular epithelial cells. Thus, caspase 11 promotes Panx1 cleavage to stimulate channel activity and maturation of IL-1β (Yin et al., 2022). Although Panx1 channels are generally recognized as essential actors in NLRP3 inflammasome activation, NLRP3 signaling can be Panx1-independent. In particular, knockdown and overexpression of Panx1 proteins does not affect NLRP3 inflammasome activation in THP-1 cells upon lipopolysaccharides (LPS) stimulation (Wang et al., 2013). Further experiments with *Panx1* knock-out mice showed that Panx1 proteins are not required for NLRP3 inflammasome activation (Wang et al., 2013; Chen et al., 2020). In addition, Panx1 channels associate with NLRP1 signaling to trigger caspase 1. High extracellular potassium concentrations induce Panx1 channel opening and caspase 1 activation in rat cortical neurons and astrocytes (Silverman et al., 2009). Panx1 channel opening is equally triggered by the AIM2 inflammasome pathway. Blocking Panx1 channel-mediated communication reduces the secretion of pro-inflammatory cytokines IL-1β and IL-18 in rats following AIM2 inflammasome activation (Zheng et al., 2022).

##### 2.5.1.2 Activation of immune cells

The pro-inflammatory role of Panx1 channels is underscored by their ability to modulate inflammatory responses through immune cell stimulation. Murine adipocytes and aortic endothelial cells express functional Panx1 channels that facilitate extracellular release of ATP molecules (Tozzi et al., 2020; Filiberto et al., 2022). Functional Panx1 channels promote macrophage migration (Tozzi et al., 2020) and activation, by triggering P2X7 receptors, and increasing the secretion of IL-1β (Filiberto et al., 2022). Likewise, activated Panx1 channels in murine T lymphocytes stimulate P2X receptors in an autocrine way to mediate the production of IL-2 and the proliferation of T lymphocytes (Schenk et al., 2008). P2X1/P2X4 receptor-mediated activation of *IL2* gene transcription is also observed in human peripheral blood mononuclear cells and mouse splenocytes upon Panx1 channel-induced ATP release (Woehrle et al., 2010). Additionally, the interplay between purinergic receptors and Panx1 proteins underlies macrophage maturation. In order to form multinucleated macrophages, P2X7 receptor-mediated ATP release and Panx1 protein expression are essential, as shown in murine peritoneal macrophages stimulated with the inflammatory cytokine macrophage colony-stimulating factor (Lemaire et al., 2011). Furthermore, leukocyte activation is associated with Panx1 channel signaling. Panx1 channel-mediated ATP release evokes adhesion and migration of leukocytes towards inflamed tissue in mice (Lohman et al., 2015). Moreover, Panx1 channel activity is important for chemokinesis processes. The opening of Panx1 channels in epithelial cells is required to control ATP-induced chemokinesis in rat epiplexus cells (Maslieieva and Thompson, 2014).

#### 2.5.2 Cell death

##### 2.5.2.1 Apoptosis

**FIGURE 4:**
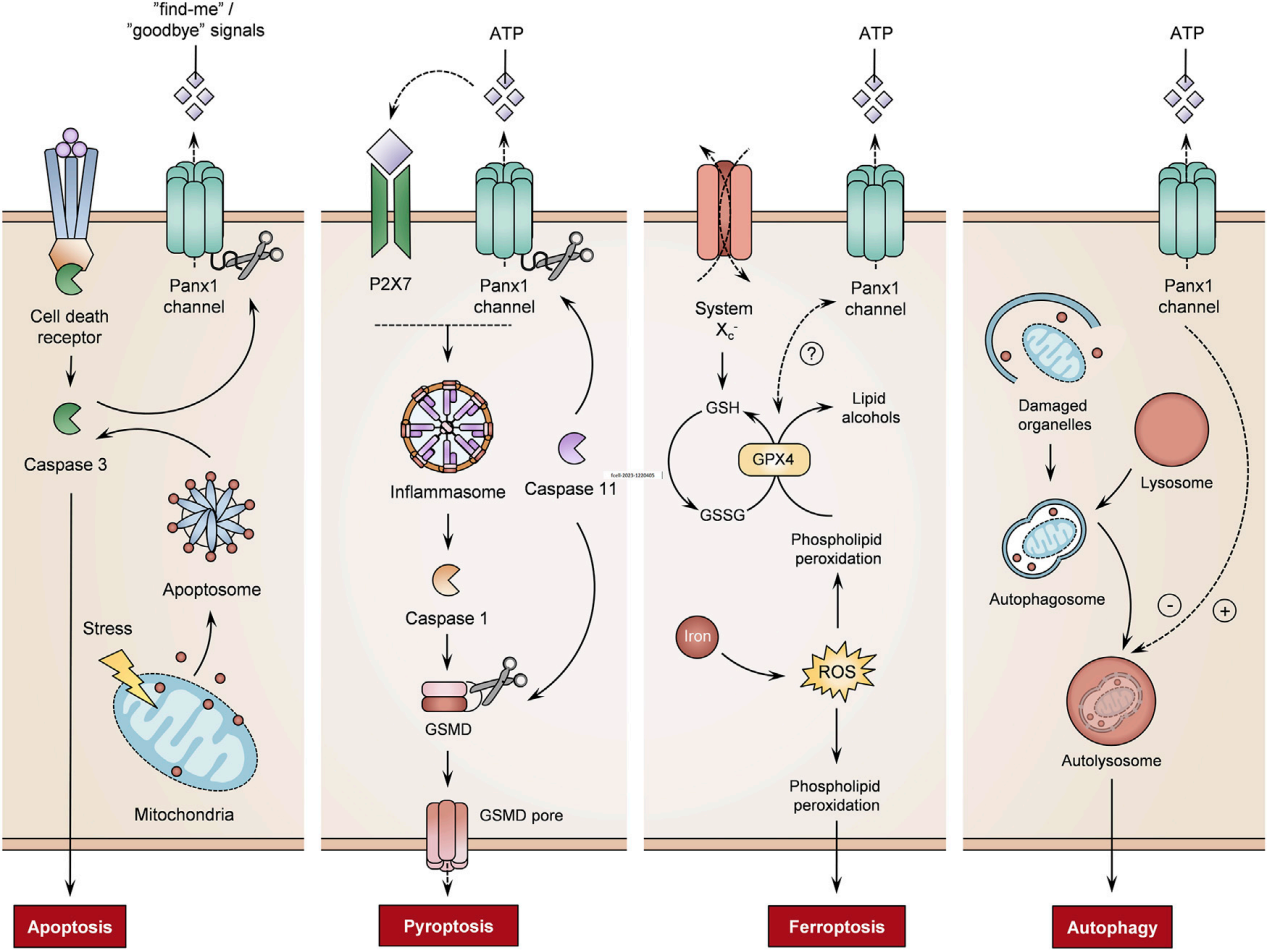
Role of pannexin1 (Panx1) channels on different cell death mechanisms. Panx1 channel activity controls the regulation of cell death processes, including apoptosis, pyroptosis, ferroptosis and autophagy (adenosine triphosphate (ATP) molecules, gasdermin D (GSMD), glutathione peroxidase 4 (GPX4), oxidized glutathione (GSSH), purinergic receptor 7 (P2X7), reactive oxygen species (ROS), reduced glutathione (GSH)) (+ = induction, - = inhibition, ? = not yet fully understood).

Apoptosis is an energy-dependent process of programmed cell death characterized by various morphological changes, such as chromatin condensation, nuclei fragmentation and cell plasma membrane blebbing. During apoptosis, the cellular content is sealed in apoptotic bodies, which are removed by macrophages (Battistelli and Falcieri, 2020). Panx1 proteins are instrumental in this process, in particular in the activation of macrophages (Qu et al., 2011). Furthermore, Panx1 channels play an important role in apoptosis by releasing “find-me” signals (Figure 4). Panx1 channel-mediated signaling controls the recruitment of monocytes in apoptotic human T lymphocytes. Panx1 channel activity is hereby triggered by caspase cleavage (Chekeni et al., 2010). On the other hand, Panx1 channels release metabolites as “goodbye” signals (Figure 4) that modulate multiple gene programs in neighboring cells. By doing so, the opening of Panx1 channels in apoptosis may influence surrounding tissue (Medina et al., 2020). In this way, Panx1 channels establish neutrophil extracellular traps in apoptotic human breast cancer cells (Mukai et al., 2022). A function for caspase-stimulated Panx1 channel activity in apoptosis has been further demonstrated in murine bone marrow-derived macrophages and apoptotic cervical cancer cells (Chen et al., 2020; Imamura et al., 2020). Furthermore, pharmacological blocking of Panx1 channels increases the formation of apoptotic bodies in human T lymphocytes undergoing apoptosis. This effect is only observed in apoptotic cells. Without an apoptotic stimulus, the inhibition of Panx1 channels does not induce apoptosis nor the formation of apoptotic bodies (Poon et al., 2014). The observation that Panx1 channels regulate fragmentation of apoptotic bodies in murine thymocytes demonstrate their involvement as regulators of cellular disassembly during apoptosis (Poon et al., 2014). Panx1 channel opening provokes the accumulation of intracellular calcium ions in rat spinal neurocytes. The overexpression of Panx1 proteins is associated with enhanced expression levels of proapoptotic markers and induction of apoptosis (Huang et al., 2021). In addition, silencing Panx1 protein expression and blocking Panx1 channel activity limit apoptosis and production of apoptosis-related proteins in cultured human renal tubular epithelial cells and mice, respectively (Huang et al., 2020). Panx1 channels also participate in chemotherapeutic drug-induced apoptosis (Wu D. et al., 2017; Boyd-Tressler et al., 2017). In particular, Panx1 channels mediate ATP release to regulate the apoptosis rate in cisplatin-treated mouse Leydig cells (Wu D. et al., 2017). On the other hand, the opening of Panx1 channels has been identified as an underlying cause of apoptosis. Inhibition of Panx1 channels prevents subsequent responses that lead to apoptosis. In this way, blocking Panx1 channels prevents apoptosis through antagonism of p38 mitogen-activated protein kinase and TLR2/4/NF-κβ signaling pathways (Wu L. Y. et al., 2017; Luo et al., 2017).

##### 2.5.2.2 Pyroptosis

Pyroptosis is an inflammatory form of lytic cell death, which is linked with inflammasome signaling. Activation of the inflammatory pathway triggers caspases that produce pro-inflammatory cytokines and pore-forming proteins. These pores allow the influx of water, leading to cell swelling and rupture of the cell plasma membrane (Yu et al., 2021). Panx1 channel activity induces pyroptosis via a P2X7-dependent mechanism (Figure 4). As such, caspase 11 cleaves Panx1 to stimulate Panx1 channel-dependent ATP release. This activates P2X7 receptors, leading to pyroptosis in murine bone marrow-derived macrophages (Yang et al., 2015). Similarly, Panx1 expression is required for pyroptosis processes in THP-1-derived macrophages (Zhang S. et al., 2021). Furthermore, experiments using the D379A human *PANX1* mutant show that the caspase cleavage site controls pyroptosis. In addition, S424A mutation in the *C*-terminus of the PANX1 channel promotes pyroptosis when human macrophages are stimulated with LPS and ATP (Zhang S. et al., 2021). Moreover, liver X receptor β activation induces pyroptosis of human and murine colon cancer cells through Panx1 channels. The liver X receptor β interacts with Panx1 to trigger ATP release, in turn inducing pyroptosis by mediating NLRP3-inflammasome-dependent activation of caspase 1 (Derangère et al., 2014). In THP-1 cells, Panx1 channels also drive NLRP3 inflammasome signaling to trigger pyroptosis (Zhou et al., 2022).

##### 2.5.2.3 Ferroptosis

Ferroptosis, an iron-dependent type of programmed cell death, is characterized by typical morphological changes, including the loss of structural integrity, shrinkage of mitochondria, a reduction or disappearance of mitochondrial cristae and rupture of the cell plasma membrane (Li et al., 2020; Jiang et al., 2021). Ferroptosis is induced through inhibition of the glutathione/glutamine antiporter system X_c_^−^, inactivation of glutathione peroxidase 4, lipid peroxidation or iron-driven generation of ROS (Figure 4) (Yang and Stockwell, 2016; Li et al., 2020). Although the link between Panx1 channel activity and ferroptosis is not yet fully understood, experiments with cultured human tubular cells and mice show that Panx1 proteins are implicated in the mediation of ferroptosis in a renal ischemia/reperfusion-related injury. *PANX1* gene transcription silencing reduces ferroptotic cell death *in vitro* and *in vivo* (Su et al., 2019). Furthermore, Panx1 expression regulates the production of ferroptosis-related proteins in human tubular cells upon exposure to erastin, an inhibitor of glutathione peroxidase 4 activity (Su et al., 2019). A similar correlation between Panx1 mRNA expression and ferroptosis-associated markers is seen in placental tissues. In preeclampsia patients, increased expression of Panx1 and pro-ferroptosis mediators has been described, while anti-ferroptosis regulators are negatively correlated (El-Khalik et al., 2022). A gene signature study on lung adenocarcinoma also identified the *PANX1* gene as a ferroptosis-related prognosis gene. The established ferroptosis-related gene signature associates the overexpression of *PANX1* with unfavorable impacts on lung adenocarcinoma prognosis (Zhang A. et al., 2021).

##### 2.5.2.4 Autophagy

Autophagy entails the process of clearing old, damaged or abnormal cellular components via lysosomal degradation. Although autophagy is generally considered a cell protection mechanism, it is also a decisive process for cell death. Autophagic cell death features a state of cell death utilizing the autophagy machinery (Denton and Kumar, 2019; Jung et al., 2020). Panx1 channel-mediated ATP release suppresses autophagy in murine testicular cancer cells (Figure 4). While the knockdown of Panx1 expression and inhibition of Panx1 channels promotes autophagy, overexpression of Panx1 leads to the release of ATP, in turn hampering the autophagy process (Yuan et al., 2022). However, Panx1 channel activity also limits autophagy (Figure 4). In a sepsis mouse model, the inhibition of Panx1 channels alleviates pyroptosis and activates autophagy to establish a neuroprotective effect (Lei et al., 2021). On the other hand, Panx1 channels introduce an inflammatory feature in autophagic cells. Mouse bone-marrow-derived B lymphocytes undergoing autophagy release ATP in a Panx1 channel-dependent manner. Phagocytosis of such autophagic cells causes inflammasome activation in mouse macrophages, as evidenced by an increase in NLRP3 signaling and secretion of IL-1β (Ayna et al., 2012).

## 3 The role of pannexin1 channels in liver disease

### 3.1 Acute liver disease

Acute liver failure (ALF) encompasses a rapid decline in liver function, *i.e.*, within days or weeks, in a patient without preexisting liver disease. ALF is characterized by jaundice, coagulopathy and hepatic encephalopathy (Weiler et al., 2020). In addition, liver function tests of ALF-patients often display a prolonged prothrombin time, elevated serum levels of bilirubin, alanine aminotransferase (ALT) and aspartate aminotransferase (AST) (Björnsson and Olsson, 2005; Weiler et al., 2020). Impairment of liver function can be evoked by many triggers, such as viruses and drugs (Fontana, 2008; Weiler et al., 2020). Although reports on the etiology and incidence of ALF vary by region, acetaminophen (APAP)-induced liver disease is the leading cause of drug-induced liver failure in Western countries (Jaeschke et al., 2020). In overdose, APAP induces hepatotoxicity through the accumulation of *N*-acetyl-*p*-benzoquinone imine (NAPQI), a metabolite of APAP. Consequently, NAPQI provokes an inflammatory response in the liver (Jaeschke et al., 2020). Panx1 channels play an essential role in the pathogenesis of liver injury triggered by APAP. Genetic deletion of *Panx1* partially prevents mice from developing APAP-induced liver damage as indicated by a decrease in serum ALT and AST activities. Application of a Panx1 channel blocker diminishes APAP-mediated liver injury as evidenced by a decrease in serum and liver levels of TNF-α, IL-6 and IL-10, reducing recruitment of neutrophils, altering the oxidative liver status and promoting liver regeneration in mice (Maes et al., 2017; Willebrords et al., 2018). Furthermore, APAP overdosing changes hepatic RNA and protein expression of Panx1 in mice. After APAP treatment, enhanced *Panx1* gene transcription and Panx1 protein levels are measured in liver tissue (Maes et al., 2017). Panx1 RNA and protein expression levels are also increased during hepatic ischemia-reperfusion in rats and mice, respectively (Kim et al., 2015; Musavi et al., 2022). Ischemia-reperfusion injury is a major cause of ALF and hampers the overall success of clinical liver transplantation. This pathological process involves hepatic cell death and inflammation-mediated liver injury. Ischemic conditions expose hepatic cells to oxygen deprivation, pH changes and ATP depletion, heightening their glycogen-dependency and leading to parenchymal cell death. Subsequently, hepatic reperfusion triggers inflammation reactions promoting hepatocellular injury in the acute phase of liver ischemia-reperfusion injury (Zhai et al., 2013; Lu et al., 2016; Hirao et al., 2022). In this regard, an NLRP3 inflammasome-mediated response is triggered during hepatic ischemia-reperfusion injury in mice. This reaction is attenuated by blocking Panx1 channel activity or through *Panx1* gene silencing and leads to a decrease in serum levels of IL-1β (Kim et al., 2015).

### 3.2 Chronic liver disease

The most prevalent chronic liver disease worldwide is a non-alcoholic fatty liver disease (NAFLD), characterized by fat accumulation in hepatocytes that is not caused by alcohol consumption (Cheemerla and Balakrishnan, 2021; Pouwels et al., 2022). The progression of NAFLD towards non-alcoholic steatohepatitis (NASH) is driven by multiple insults, including insulin resistance, hormones secreted from the adipose tissue, dietary components, gut microbiota and genetic pathways (Buzzetti et al., 2016; Caturano et al., 2021). These mechanisms trigger intense inflammatory activity in the liver, which is a hallmark of NASH. NASH-related hepatotoxicity can be experimentally induced by feeding mice a specific diet, administration of drugs or through genetic modification (Willebrords et al., 2015; Lee et al., 2019; Matthews et al., 2021). A role for Panx1 proteins in NASH has been demonstrated by feeding *Panx1* knock-out mice a methionine-choline-deficient diet. *Panx1* knock-out mice show less liver damage compared to wild-type animals, evidenced by a reduction in ALT and AST activities and decrease in expression levels of liver inflammatory markers (Willebrords et al., 2018). Upon stimulation with free fatty acids, rat liver hepatoma-derived cells undergo lipoapoptosis, which goes along with Panx1 channel-mediated ATP release. This pathophysiological ATP release may act as a proinflammatory signal and contributes to hepatic inflammation in lipotoxic liver injury (Xiao et al., 2012, 2015). Similar to NASH, enhanced hepatic Panx1 RNA expression is also seen during liver fibrosis in mice (Crespo Yanguas et al., 2018; Willebrords et al., 2018). This chronic liver pathology is featured by abnormal wound healing, and is accompanied by injury and death of hepatocytes, scar formation, oxidative stress and inflammation. Liver fibrosis is typically induced in rodents through the repeated administration of carbon tetrachloride (CCl_4_) or thioacetamide (TAA) (Crespo Yanguas et al., 2016; Bao et al., 2021). *Panx1* knock-out mice are found to be less sensitive to the effects of repeated administration of CCl_4_ (Crespo Yanguas et al., 2018). Moreover, *Panx1* knock-out animals display reduced collagen content, hepatic stellate cell activation, inflammation and hepatic regeneration. The latter might comply with an underlying mechanism of NLRP3 inflammasome activation. Mice lacking ASC and NLRP3 show less fibrosis than wild-type littermates after treatment with CCl_4_ and TAA (Watanabe et al., 2009). The direct role of NLRP3 inflammasome in liver fibrosis has also been demonstrated in other experimental models of liver fibrosis. The absence and inhibition of NLRP3 attenuate hepatic fibrosis in murine models of aging and *Schistosoma japonicum* infection, respectively (Zhang et al., 2019; Gallego et al., 2020). Moreover, Panx1 channel-directed ATP release contributes to fibrosis in mice. Thus, blocking Panx1 channels prevents the development of hepatic fibrosis in a murine model of TAA-induced fibrosis (Feig et al., 2017). In contrast, ablation of Panx1 proteins even exacerbates the inflammatory profile in a surgical-based bile duct ligation (BDL) mouse model (Crespo Yanguas et al., 2018). BDL elicits a cholestatic injury and periportal biliary liver fibrosis (Crespo Yanguas et al., 2016), but this might be independent of NLRP3-independent mechanisms as a BDL-surgery increases liver fibrosis in the absence of caspase 1 in mice (Cai et al., 2020). This is, however, still surrounded by some controversy, since it has been documented that NLRP3 inflammasome inhibition suppresses cholestatic liver fibrosis in BDL-mice (Gong et al., 2016; Qu et al., 2019; Wang and Hauenstein, 2020; Frissen et al., 2021). Furthermore, a putative role for Panx1 channels is also seen during hepatitis C-virus (HCV) infection. In human hepatoma-derived cells, Panx1 channels control the release of exosomes and microRNAs contributing to liver disease development caused by HCV infection (Kim et al., 2021). On the other hand, hepatic Panx1 channels convey protective roles in donor livers. Panx1 channel activity in the liver controls the production of IL-33 and stimulates recruitment of macrophages and neutrophils. Thus, low Panx1 expression profiles are associated with an increased risk for methicillin-resistant *Staphylococcus aureus* infection in human liver transplantation recipients (Li et al., 2021). Moreover, this Panx1-IL33 axis plays a protective role in LPS-induced endotoxemia in mice by promoting the resolution of inflammation. Hepatic Panx1 expression and associated Panx1 channel activity regulates a defense mechanism by increasing the number of liver-infiltrating regulatory T lymphocytes and attenuating the cytokine storm (Wang et al., 2022).

## 4 Discussion and future perspectives

Although Panx proteins have only been discovered about 2 decades ago, there is a general consensus regarding their role in physiological and, in particular, pathological processes. Panx1 channel activation is indeed considered as a key event in inflammation and cell death. A generic mechanism for the onset of inflammation and cell death could involve Panx1 channel-mediated extracellular ATP release. This trigger is involved in a plethora of diseases, including liver pathologies. However, few reports describe a protective role for Panx1 proteins. Indeed, genetic deletion of *Panx1* is associated with dysfunctionalities in multiple organs. Future studies should pinpoint whether Panx1 protein expression or Panx1 channel activity controls the regulation of inflammatory reactions and cell death processes. The Panx research domain strongly relies on Panx1 channel blockers to elucidate (patho) physiological functions of Panx1 channels. However, established Panx1 channel inhibitors lack selectivity, specificity, potency and/or stability (Willebrords et al., 2017; Caufriez et al., 2020; Van Campenhout et al., 2021; Koval et al., 2023). New quinoline-based Panx1 channel inhibitors show great promise with their efficiency and structural relevance for channel blocking. However, more *in vivo* studies must confirm their efficiency and safety (Crocetti et al., 2022; Crocetti et al., 2023). Nevertheless, Panx1 channel blockers show great potential as anti-inflammatory drugs (Willebrords et al., 2017; Caufriez et al., 2020; Van Campenhout et al., 2021). Several groups are therefore focusing on the development of new Panx1 channel inhibitors. In line with this, the artificial intelligence (AI)-assisted drug discovery industry is gaining more attention. To date, numerous AI-based computer programs are applied for *de novo* drug design (Paul et al., 2021). Although this AI-driven approach is promising, it should be emphasized that the classic research path for drug discovery remains relevant. Thus, existing compound libraries show tremendous potential. High-throughput screening of natural product libraries should be embraced in the search for novel Panx1 channel blockers. Silibinin, a plant-derived polyphenol, protects hepatocytes against ischemia-reperfusion injury by suppressing mRNA expression of Panx1 in the liver (Musavi et al., 2022). In line with this, various screening strategies and functional assays can be developed to select for specific compound characteristics, such as affinity, specificity or blocking potential. Indeed, the identification of Panx1 channel inhibitors is a technical challenge. A number of tools exist to study the functionality of Panx1 channels, yet they are based on the measurement of variable and non-specific outputs, such as conductance states, extracellular release of ATP or cytosolic uptake of tracer dyes (Koval et al., 2023). In addition, progress has been made over the last few years with the establishment of Panx1 cryo-electron microscopy data (Deng et al., 2020; Qu et al., 2020; Zhang S. et al., 2021). This data suggest Panx1 channels be heptameric (Qu et al., 2020) instead of hexameric (Boassa et al., 2007), as initially predicted. However, the structure of the ATP-release channel is unknown. Encouraging research in this direction will open perspectives in the search for (hepatic) Panx1 channel blockers as it allows to determine how a Panx1 channel inhibitor interacts with its target, *i.e.*, whether it sterically blocks the channel or induces a conformational rearrangement leading to channel closure.

## Abbreviations

AI, artificial intelligence; ALF, acute liver failure; ALT, alanine aminotransferase; APAP, acetaminophen; ASC, apoptosis-associated speck-like protein containing caspase; AST, aspartate aminotransferase; ATP, adenosine triphosphate; BDL, bile duct ligation; CCl4, carbon tetrachloride; CREB, 3′,5′-cyclic adenosine monophosphate response element-binding protein; DAMPs, damage-associated molecular patterns; ETV4, E26 transformation-specific translocation variant 4; HCV, hepatitis C-virus; ILs, interleukins; LPS, lipopolysaccharides; NAFLD, non-alcoholic fatty liver disease; NAPQI, N-acetyl-p-benzoquinone imine; NASH, non-alcoholic steatohepatitis; NF, nuclear factor; NLRP3, NOD-like receptor family pyrin domain containing 3; PAMPs, pathogen-associated molecular patterns; Panx, pannexin; TAA, thioacetamide; TLRs, Toll-like receptors; TNF, tumor necrosis factor.
